# Flexible embryonic shell allies large offspring size and anti-predatory protection in viviparous snails

**DOI:** 10.1038/s41598-022-22651-w

**Published:** 2022-10-25

**Authors:** A. Sulikowska-Drozd, T. K. Maltz, K. Janiszewska

**Affiliations:** 1 Department of Invertebrate Zoology and Hydrobiology, University of Lodz, Lodz, Poland; 2 Museum of Natural History, University of Wrocław, Wrocław, Poland; 3 Institute of Paleobiology, Polish Academy of Sciences, Warsaw, Poland

**Keywords:** Ecology, Evolution, Zoology

## Abstract

The evolutionary conflicts between viviparous reproductive mode and skeleton shape may occur whenever the space available for embryo development or delivery is limited by hard inflexible structures of a parent (bones, shell, etc.). In tetrapods, offspring size is at odds with female locomotion efficiency, which results in obstetric selection. We suggest a similar relationship for viviparous gastropods, where spacious canal needed for embryo delivery may interfere with anti-predatory role of narrow and toothed shell aperture. We explored this hypothesis in the group of viviparous land snails (Clausiliidae, subfamily Phaedusinae), known for complex apertural barriers protecting the shell interior. Most of the shell structure modifications we recorded facilitate the delivery of embryos but simultaneously reduce the safeguard of a narrow shell opening. However, we also observed highly flexible embryonic shells that may withstand squeezing between apertural barriers during birth. We investigated the microstructure of these flexible embryonic shells, compared to the typical hard shells of clausiliid embryos, which are rigid and unpliable already in the genital tract of the parent. Our results suggest that the unusual flexibility, which is related to a low number of organomineral layers in the shell, evolved in two phylogenetically distant lineages of Phaedusinae. This adaptation reduces mechanical constraints for birth of the neonates but allows to maintain the protective function of the apertural barriers.

## Introduction

Two antagonistic selection pressures occur regarding offspring size and maternal morphology in humans: the first, for a larger birth canal, permitting successful passage of a large-brained neonate, and the second, for smaller pelvic dimensions, required for bipedal locomotion^1,2^. The evolutionary compromise between these opposing selective mechanisms, often called obstetric selection, has led to several adaptations that reduce maternal and perinatal mortality from obstructed labour. Among them, the curved, multirotational trajectory of the fetus through the maternal pelvis, flexion of fetal head, and open fontanelles are the most important. Yet, a tight fit between the dimensions of the birth canal and those of the brain and body of the offspring is maintained even in a changing environment by the offspring matching its growth trajectory to metabolic signals of the maternal phenotype^3^.

Trade-offs which resemble obstetric selection may function in any animal with a skeleton which limits the space available for embryo development or hinders their delivery through a narrow, relatively inflexible passage. For example, in tetrapods, a pelvic constraint model convincingly explains a functional upper size limit for progeny both in live-bearing and egg-laying species^4–6^. Another group of animals most likely experiencing some kind of obstetric selection are shelled gastropods. However, the studies regarding the selection on size and shape, which are related to reproduction mode, are very rare in snails^7^.

The gastropod shell is an example of an external skeleton that also functions as a defence against various external threats, such as predation or desiccation in terrestrial environments. This skeleton always has its most vulnerable spot, the aperture, through which soft body parts pass out of the shell whenever the animal is moving or feeding. The smaller the area of the aperture, the better the protective function of the shell, thus additional structures constricting the area of the aperture, such as apertural barriers (e.g., teeth or folds), have evolved in many land snail families^8^. The teeth probably deter small-sized predators that try to enter the shell but there are also other hypotheses that explain their importance^8,9^. In any case, these structures may interfere with the reproductive strategy of snails^10^. Gastropods with strong apertural barriers produce non-calcified or partly calcified eggs^11^. Such eggs are pliable and can be compressed during passage through the aperture and regain their original shape afterwards. In contrast, gastropods with a wide aperture without teeth can produce eggs with low shape flexibility, i.e. heavily calcified eggs. Similarly, in viviparous snails, we expected a strong selection towards a wide aperture and a smooth internal surface of the shell canal, which provide less mechanical constraint for passing of the shelled neonates^10^.

The trade-off between antagonistic selection pressures imposed by reproductive traits may affect a large number of land snail taxa, because viviparity (previously called ovoviviparity) occur in at least 30 families of stylommatophorans, hermaphroditic land snails^12,13^. On the other hand, the great diversity of shell geometry limits the distribution of the acute examples of obstetric selection to snails with a relatively small shell mouth or strong aperture barriers that can obstruct the passage of embryo. This kind of morphology is typical for Clausiliidae, known for their elaborate apertural barriers^14^ and containing many viviparous or embryo-retaining species^15^.

Clausiliid shells are high, slender and usually fusiform or club-shaped. They consist of many whorls (often 10–12). On the interior surface of the body whorl they possess a complex closing apparatus (clausiliar) that includes several narrow folds known as lamellae and plicae, and a single calcareous plate (clausilium), connected to the shell columella via a flexible stalk^14^. The shape of the clausilium plate, especially its width, corresponds well to the cross-cut of the shell canal at its narrowest point. Although the main functions of apertural barriers are debatable^10,16^, it is obvious that the clausilium blocks the passage to a shell canal when the snail retracts its body, and thus may confer an antipredator benefit. When a snail becomes active, its head and foot pass between folds of the apertural barriers and push the clausilium aside to emerge from the shell. During parturition, the shelled neonate has to pass through the same constriction (a genital opening is situated on the left side of the snail’s head), which implicates opposing selective pressures operating on embryo size and size of the shell canal^7^. Considering these relationships, it is intriguing that clausiliid snails with well-developed apertural barriers, have acquired a viviparous reproductive mode and produce offspring that are relatively large in comparison to the aperture of the adult^15^.

Viviparous species belong to several subfamilies of Clausiliidae, yet the strategy seems to be most frequent in the subfamily Phaedusinae. Recently, the reconstruction of the molecular phylogeny of the subfamily revealed that the basal taxa of the group are oviparous, while viviparity has evolved several times independently^17^. Besides the 21 viviparous or embryo-retaining taxa included in this study, more than 70 viviparous species have been reported in the subfamily^18–20^. The discovery, that viviparity evolved repeatedly in the Phaedusinae subfamily, each time overcoming the mechanical constraints of apertural barriers, makes this family an interesting model group for studying obstetric selection in gastropods.

As the molecular data suggest a repeated and independent evolution of viviparity among the Phaedusinae, we suppose that constraints associated with the live-bearing strategy may have been overcome in a variety of ways. In this paper, we investigate a handful of adaptations that allow for the passage of the embryo through the occlusion of apertural barriers in the parental shell. Some of these were mentioned briefly in taxonomic papers^21^, and we review these and illustrate them for comparative purposes in several clausiliid species: *Reinia* *variegata* (A. Adams, 1868), *Reinia* *ashizuriensis* Azuma, 1968, *Stereophaedusa* *addisoni* (Pilsbry, 1901), *Tauphaedusa* *sheridani* (L. Pfeiffer, 1865), *Euphaedusa* *steetzneri* (Pilsbry, 1919), *E.* *cylindrella* (Heude 1886). The main goal, however, is to present a novel adaptation concerning the structure of the embryonic shell found in *Oospira* *miranda* (Loosjes & Loosjes-van Bemmel, 1973) and *Zaptyx* *ventriosa* (Schmacker & Boettger, 1891), that was previously entirely overlooked.

## Results

The studied viviparous clausiliids developed four types of morphological adaptations that facilitate the delivery of embryos through the shell aperture: (1) reduction of the clausiliar apparatus, (2) decrease of embryonic shell width, (3) widening of the shell canal, and (4) development of a flexible embryonic shell.

### Reduction of the clausiliar apparatus

**Figure 1:**
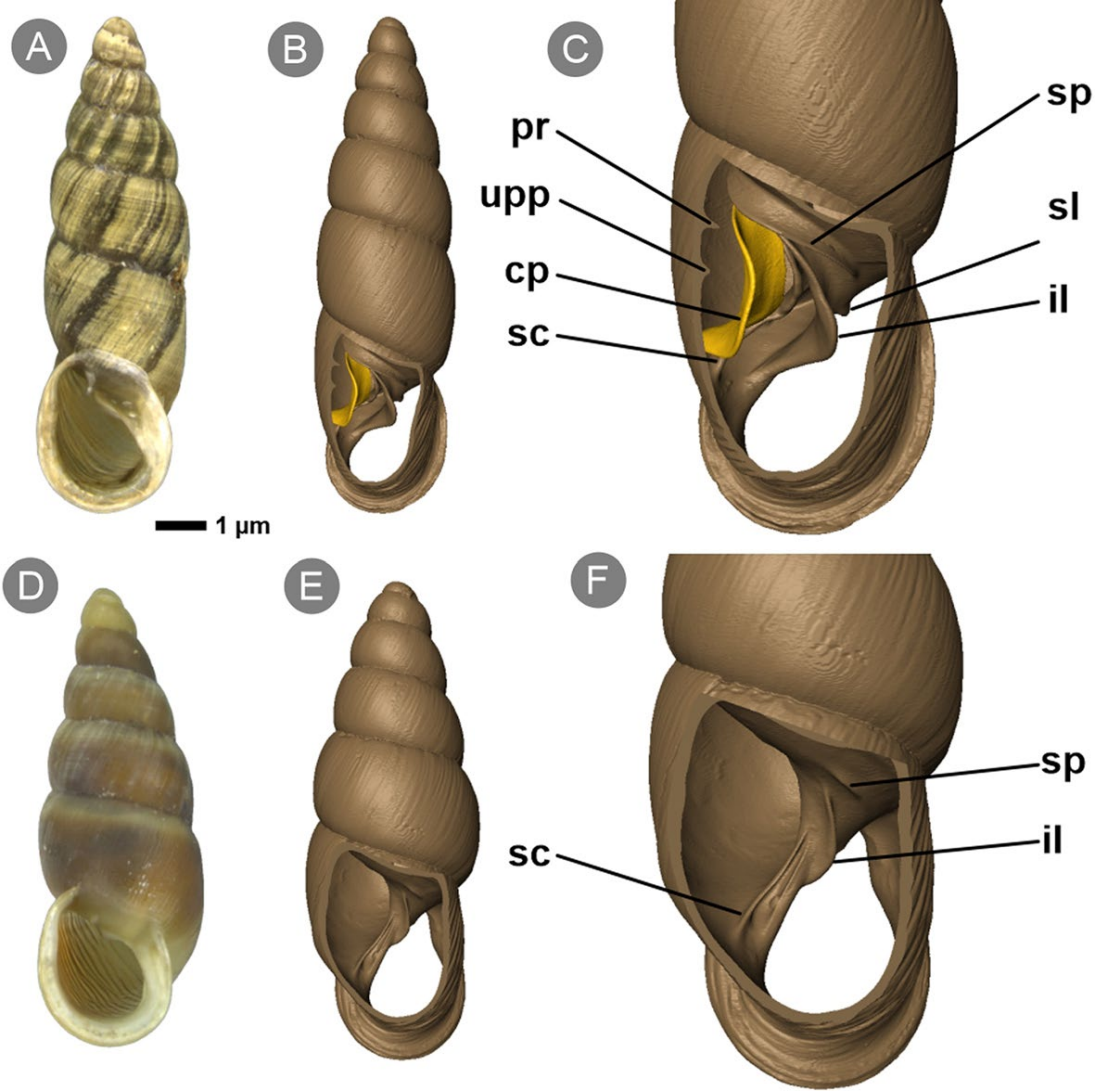
Different stages of reduction of apertural barriers in members of genus *Reinia*: *R.* *ashizuriensis* (**A–C**; upper row) and *R.* *variegata* (**D–F**; lower row). (**A,D**) Adult shells; (**B,C,E,F**) adult shells with body whorl cut open dorsally in microCT visualisation. *cp* clausilium plate, *il* inferior lamella, *pr* principal plica, *sc* subcolumellar lamella, *sl* superior lamella, *sp* spiral lamella, *upp* upper palatal plica.

Members of the *Reinia* genus, arboreal species from Japan (Fig. 1), show the most advanced adaptations to live-bearing compared to hypothetical ancestral Phaedusinae. The shell shape in these species is more conical than fusiform, the number of whorls decreases, and the aperture widens. One of the species, *R.* *variegata*, features almost full reduction of the clausiliar apparatus that consists of only vestigial folds (Fig. 1F). This species also lacks the clausilium, so the entrance through the aperture is unprotected.

### Decrease of embryonic shell width

**Figure 2:**
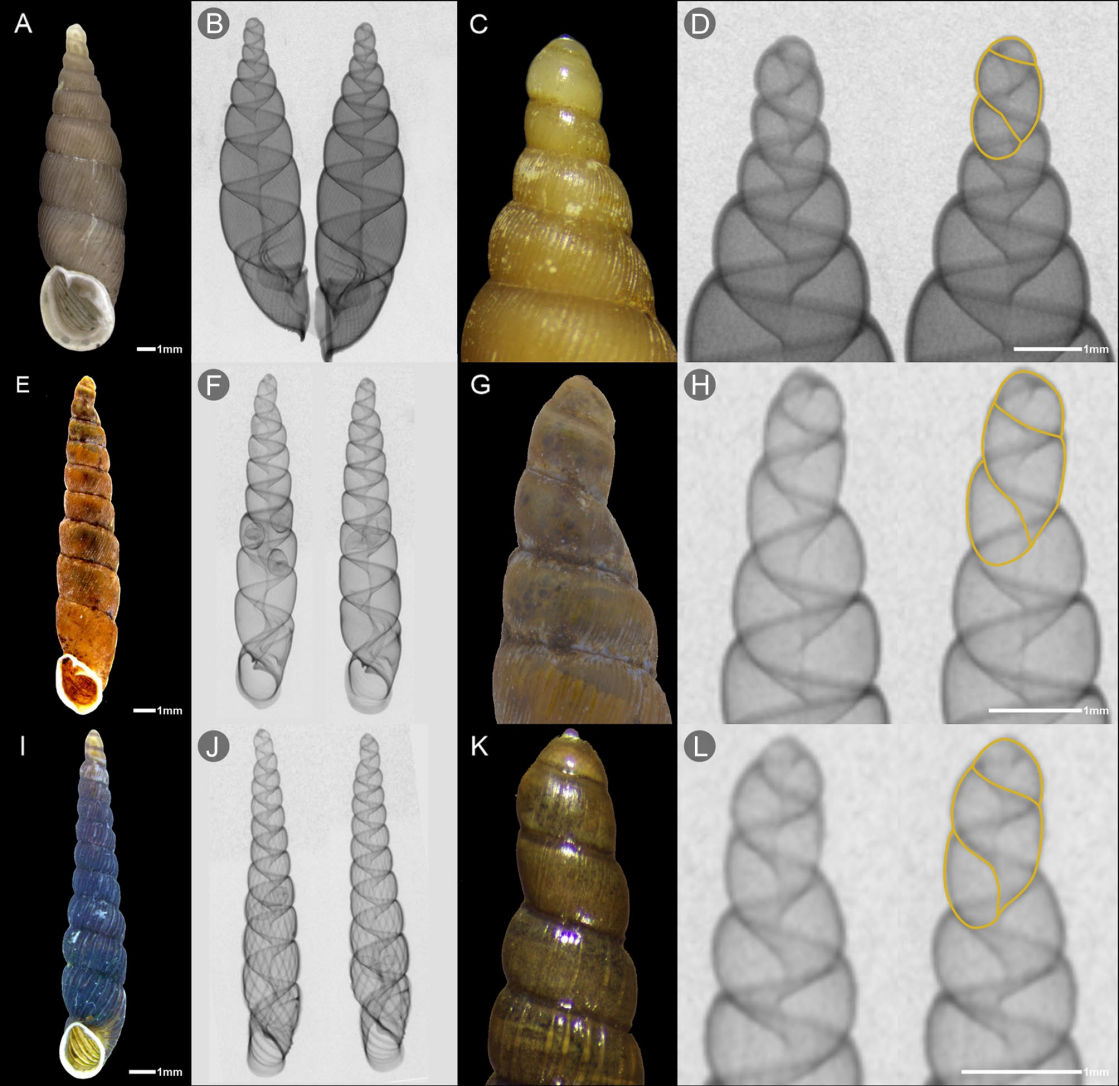
Width difference between protoconch and teleoconch in *Stereophaedusa* *addisoni* (**A–D**, upper row), *Euphaedusa* *cylindrella* (**E–H**, middle row), *Euphaedusa* *steetzneri* (**I–L**, lower row). (**A,C,E,G,I,K**) Adult shells with very narrow apical whorls; (**B,F,J**) X-rayed adults; (**F,J**) with retained embryos inside; (**D,H,L**) X-rays of apical part of adult shell with schematic drawings of a neonate.

Another adaptation concerns the shape of the embryonic shell (“protoconch”), which becomes very narrow in some viviparous species. This feature is conspicuous because embryonic whorls remain in the adult shell as apical whorls. For instance in *S.* *addisoni* (Fig. 2A–D), the apical part being much narrower than the first whorls of the teleoconch is a clear evidence that the growth trajectory has changed abruptly after birth. Other examples include *E.* *cylindrella* and *E.* *steetzneri*, in which both the protoconch and the teleoconch are very narrow, yet at the borderline between these parts, the shell axis is slightly bent (Fig. 2E–L). We suppose that this feature develops as a result of obstruction during birth.

### Widening of the shell canal

**Figure 3:**
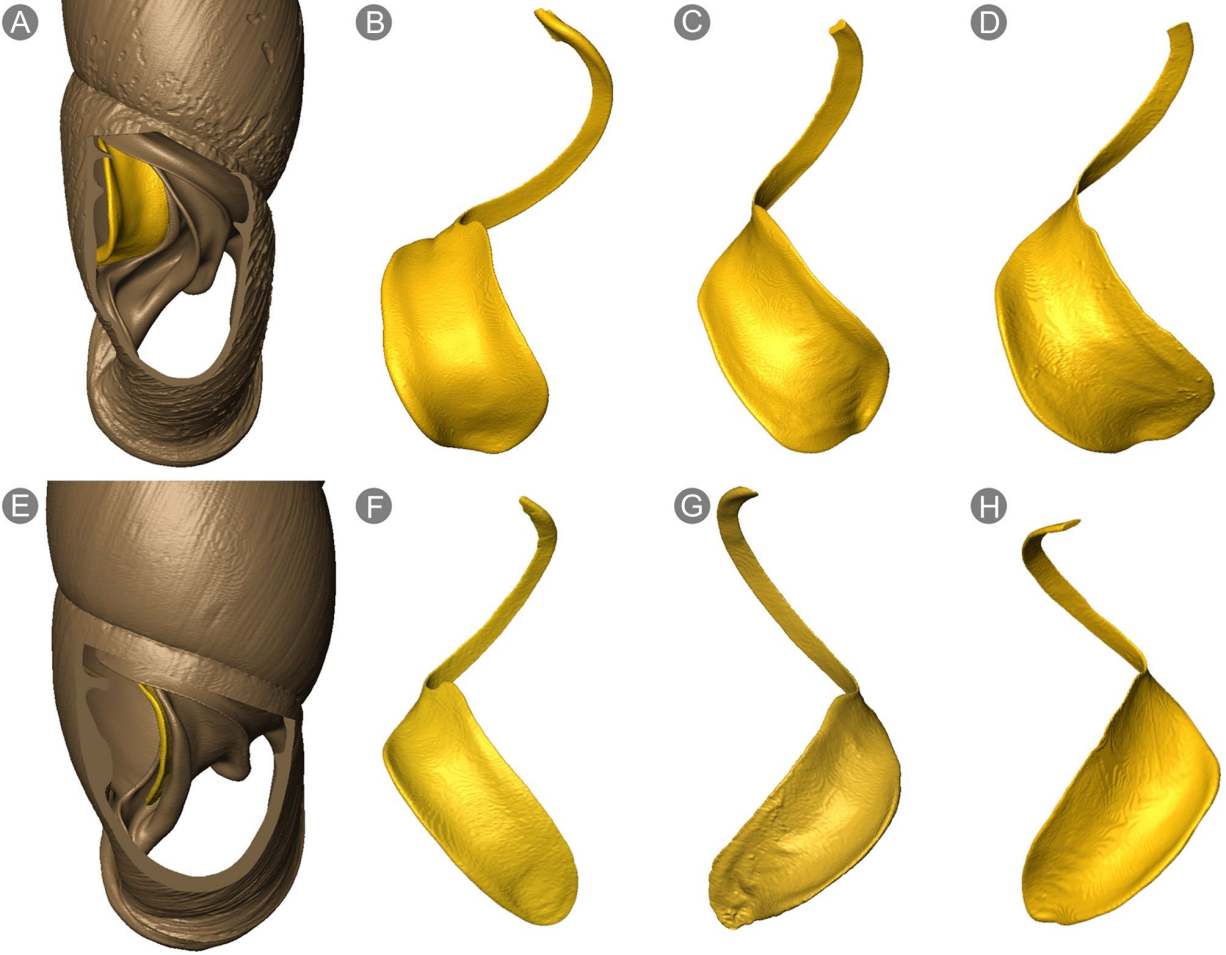
Two types of clausiliar apparatus occurring in Phaedusinae in microCT visualisation: with spirally ascending inferior lamella and wide clausilium plate (upper row), and with straight ascending inferior lamella and narrow clausilium plate (lower row). (**A**) *T.* *sheridani* adult shell with the body whorl cut open dorsally; (**B**) clausilium of *T.* *sheridani*; (**C**) clausilium of *S.* *addisoni*; (**D**) clausilium of *R.* *ashizuriensis*; (**E**) *Zaptyx* *ventriosa* adult shell with body whorl cut open dorsally; (**F**) clausilium of *Z.* *ventriosa*; (**G,H**) clausilia of *O.* *miranda.* Note, that all depicted species are viviparous.

The third type of adaptation is the widening of the shell canal in the body whorl, allowing for easier passage of the embryo between the lamellae and plicae of the apertural barriers. In this case, the outline of the shell changes only slightly giving the body whorl a more convex appearance. A substantial difference to egg-laying species concerns the apertural barriers: the clausiliar includes a broad clausilium plate and a spirally ascending inferior lamella (Fig. 3A–D). These modifications result in a spacious shell canal in the body whorl, for example in *S.* *addisoni* and *E.* *sheridani,* that can accommodate the transfer of a large embryo. Table 1 presents neonatal size in these species (shell width ca. 1.2 mm), which is very similar to their clausilium width (ca. 1.1–1.2 mm).

**Table 1:** Shell size of studied Phaedusinae species.

Species name	Adult	Adult	Adult	Adult	Neonate	Neonate	Neonate	Number of measured neonates	Clausilium plate
	SH, mean ± SD mm (range)	SW, mean ± SD mm (range)	NW, mean ± SD whorls (range)	Number of ind	SH, mean ± SD mm (range)	SW, mean ± SD mm (range)	NW, mean ± SD whorls (range)	Number of ind	Width (mm)
*Tauphaedusa* *sheridani*	14.75 ± 0.94 (13.4–17.3)	3.44 ± 0.15 (3.6–3.9)	9.61 ± 0.30 (9.0–10.3)	8	2.46 ± 0.17 (2.1–2.8)	1.26 ± 0.08 (1.1–1.5)	2.41 ± 0.23 (2.0–3.0)	37	1.101
*Reinia* *variegata*	8.50 ± 0.44 (8.0–8.8)	2.97 ± 0.06 (2.9–3.0)	5.30 ± 0.26 (5.0–5.5)	36	2.3 ± 0.15 (2.0–2.6)	1.52 ± 0.07 (1.4–1.7)	1.98 ± 0.17 (1.8–2.3)	30	–
*Reinia* *ashizuriensis*	9.52 ± 0.44 (8.7–10.0)	2.8 ± 0.09 (2.6–3.0)	6.13 ± 0.25 (5.8–6.5)	14	2.11 ± 0.15 (1.7–2.4)	1.32 ± 0.07 (1.1–1.5)	1.83 ± 0.20 (1.5–2.3)	24	0.897
*Euphaedusa* *steetzneri*	13.55 ± 0.83 (11.9–14.6)	2.52 ± 0.10 (2.4–2.6)	11.5 ± 0.5 (10.5–12.3)	6	1.54 ± 0.18 (1.2–1.8)	0.80 ± 0.08 (0.6–0.9)	2.25 ± 0.14 (2.0 – 2.5)	4	–
*Oospira* *miranda*	23.74 ± 1.53 (21.7^a^–26.4)	7.99 ± 0.45 (7.4–8.8)	7.23 ± 0.61 (5.5^a^–7.7)	11	5.19 ± 0.87 (3.4–6.7)	3.59 ± 0.36 (2.8–4.0)	2.60 ± 0.36 (2.0–3.4)	28	1.973
*Stereophaedusa* *addisoni*	16.79 ± 0.62 (15.4–17.7)	4.38 ± 0.20 (4.0–4.7)	8.43 ± 0.21 (8.1–8.7)	10	2.49 ± 0.15 (2.1–2.8)	1.25 ± 0.05 (1.2–1.4)	2.1 ± 0.17 (1.8–2.3)	31	1.234
*Zaptyx* *ventriosa*	16.1 ± 0.55 (15.2–17.2)	4.8 ± 0.19 (4.5–5.2)	7.5 ± 0.21 (7.1–8.0)	22	3.37 ± 0.34 (2.7–3.9)	2.51 ± 0.14 (2.2–2.7)	2.14 ± 0.23 (1.8–2.5)	25	1.084

Measurements based on adult individuals kept in laboratory colonies and freshly produced juveniles.

Width of clausilium plate measured in virtual microCT models.

*SH* shell height, *SW* shell width, *NW* number of whorls.

^a^Decollated adult.

Most viviparid clausiliids develop one of these three types of modification; some adaptations co-occur within a single species, for example a wide clausilium accompanies a narrow apex. Interestingly, the *Reinia* genus includes taxa with a gradual escalation of viviparity-related adaptations: *R.* *ashizurensis*, with a stout shell shape and a low number of whorls, has fully developed apertural barriers with a broad clausilium plate (Fig. 1A–C), while its congener, *R.* *variegata,* has reduced apertural barriers (Fig. 1D–F).

### Development of a flexible embryonic shell

The fourth type of adaptation found in Phaedusinae concerns the structure of the embryonic shells. We report this adaptation in *O.* *miranda* and *Z.* *ventriosa.*

**Figure 4:**
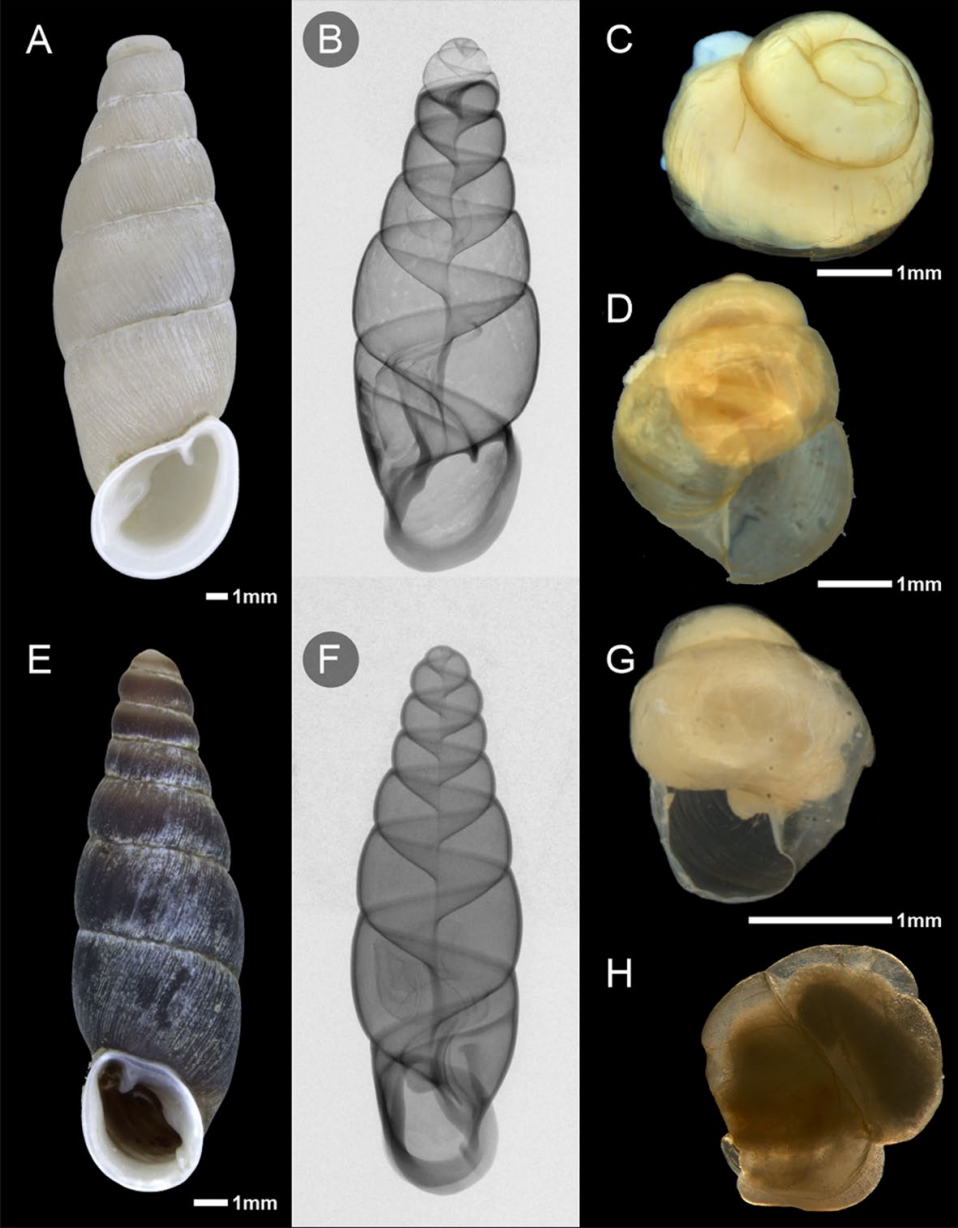
Viviparous clausiliids and their ‘soft-shelled’ neonates born in laboratory culture. (**A–D**) *O.* *miranda*: adult shell, X-rayed shell with embryo visible inside, neonates; (**E–H**) *Z.* *ventriosa*: adult shell, X-rayed shell with eggs visible inside, neonates.

*Oospira* *miranda* is a dextral, often decollated, ground-dwelling species from Vietnam (Fig. 4A). The species is viviparous: during microCT scanning of museum specimens, we found embryos within a parental shell (Fig. 4B); in laboratory culture, we observed neonates immediately after live birth (Fig. 4C,D). Morphological characters recognized in the adult shell, i.e., a wide apex (= wide embryonic shell), straightly ascending inferior lamella, and a narrow clausilium plate (Fig. 3G,H), seemed to exclude the possibility of live-bearing reproduction, as embryos are too large to pass through the shell canal at the narrowest point. The height and width of the neonatal shell (mean values: 5.19 mm, 3.59 mm) evidently exceeds the width of the clausilium plate in this species (1.97 mm) (Table 1). However, under closer examination, we found the shell to be thin and delicate, which we refer to as a ‘soft shell’. In direct examination, the neonatal shell of *O.* *miranda* resembles cellophane, which may keep a given shape for a long time but becomes distorted already under slight pressure.

A similar adaptation exists in *Z.* *ventriosa,* a Taiwanese species with a very wide apex, never decollated, a straight ascending inferior lamella, and a narrow clausilium plate (Figs. 3E,F, 4E,F). This species produces neonates in laboratory culture (Fig. 4G–H). The dimensions of the neonates (mean values: height 3.37 mm, width 2.51 mm) exceed at last twofold the width of the clausilium plate (1.08 mm). The shells of such freshly delivered juveniles, when gently touched with laboratory tweezers, became dented, but not fractured. More intense and stronger pressing can break this dentation.

These initial observations, that we made during the maintenance of the laboratory culture, suggested that the neonatal shells of *O.* *miranda* and *Z.* *ventriosa* have flexible walls. These ‘soft-shells’ seem to be highly malleable during the entire embryonic development period and delivery through apertural barriers, hardening shortly after birth. We further investigated the physical properties of the embryonic shell by means of microcomputed tomography and scanning electron microscopy.

### Microcomputed tomography

**Figure 5:**
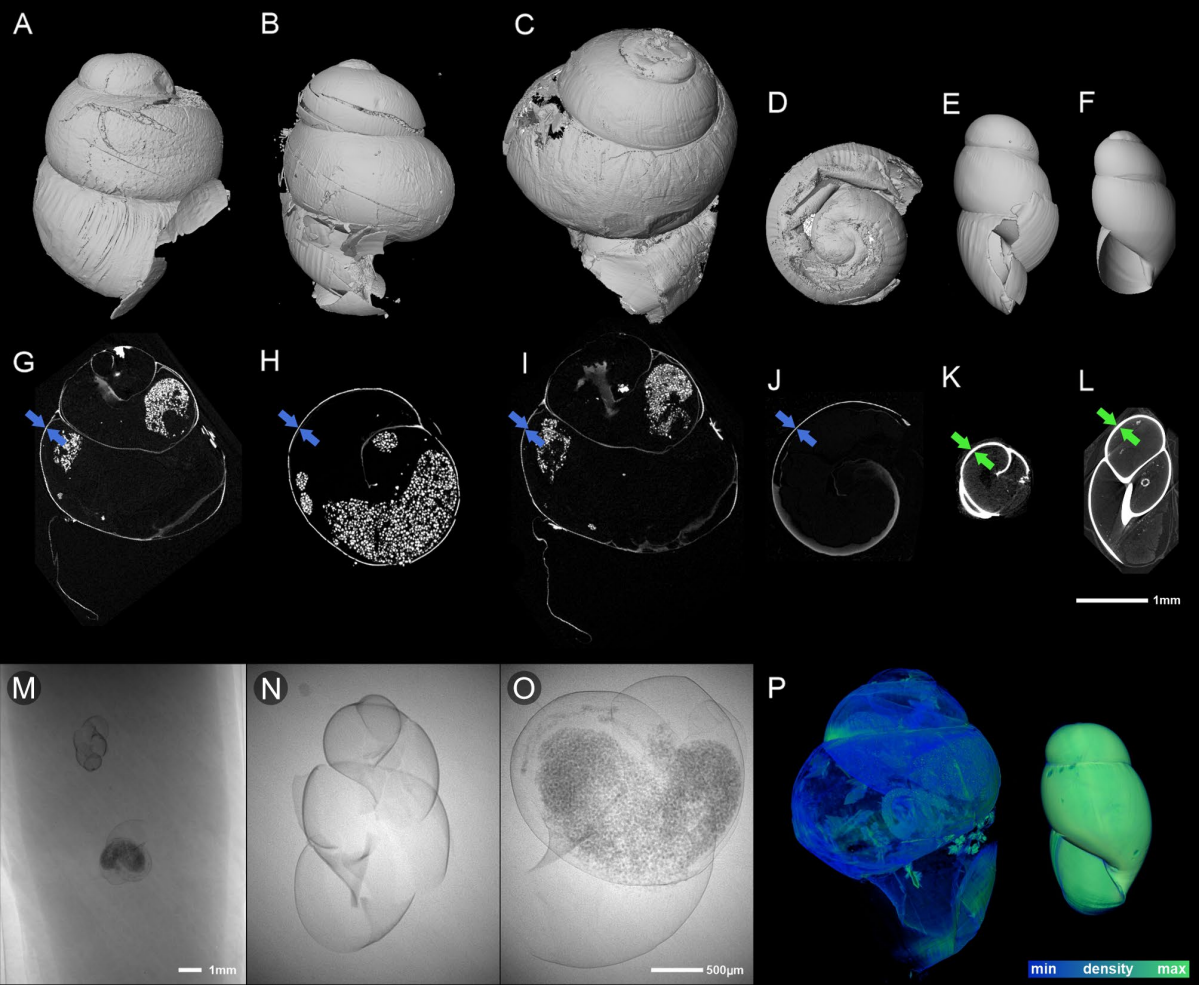
Comparison of embryonic shell thickness in clausiliids: ‘soft-shelled’ neonates of *Z.* *ventriosa* (**A,B,G,H**) and *O.* *miranda* (**C,D,I,J**); “hard-shelled” neonate of *S.* *addisoni* (**E,K**) and embryo of *T.* *sheridani* (**F,L**) scanned inside a parental shell. Upper row—microCT visualisation of shell surface; middle row—microCT sections of those specimens; (**M–O**) X-ray photographs of *S.* *addisoni* (embryo from dissected adult) and *Z.* *ventriosa* (neonate) enlarged in (**N,O**), respectively, showing the difference in shell density and thickness; (**P**) microCT based volume rendering of *O.* *miranda* (left) and *S.* *addisoni* (right) neonates, showing difference between relative density of their shells.

We scanned ‘soft-shelled’ neonates of *O.* *miranda* and *Z.* *ventriosa*, together with ‘hard-shelled’ embryos and neonates of *S.* *addisoni* and *T.* *sheridani*, in order to compare the density and thickness of the shells (Fig. 5).

Preliminary observations using the two-dimensional X-ray photographs showed a difference in thickness and density between *S.* *addisoni* and *Z.* *ventriosa* (Fig. 5M, enlarged in N and O, respectively). The 3D visualization of *O.* *miranda* and *S.* *addisoni* (the same microCT scanning and reconstruction parameters) confirmed the difference between density and shell thickness of these two species (Fig. 5P).

Due to variations in wall thickness within the neonatal shell (e.g., between the first and the second whorls), it is not possible to precisely determine the thickness of the shell wall. The accuracy of the measurement is also limited by the resolution of the microCT scans, especially in the case of the relatively large neonates of *O.* *miranda* and *Z.* *ventriosa*. When scanning the whole embryonic shell of *Z.* *ventriosa* (approximately 3.5 mm in height), the size of the voxel was approximately 1 µm. Thus, we cannot determine the shell thickness down to the nearest micron, but we can estimate it from a few to a dozen microns. A direct comparison between virtual microCT sections of specimens scanned under the same conditions shows a clear difference between the ‘soft-shelled’ and ‘hard-shelled’ taxa (Fig. 5G–L). The ’hard-shelled’ neonates have a shell wall of 30–40 µm thick. We examined the sequence of three ’soft-shelled’ *O.* *miranda* specimens that differed in size (the exact time of birth of each of the cultured neonates is unknown, ca. 1–2 days). The larger (older) the neonate was, the thicker the shell. The shell of the largest of the studied *O.* *miranda* was up to 20 µm thick. However, the shell wall of this relatively large juvenile (several millimeters in height) still did not reach the thickness of the small ’hard-shelled’ *T.* *sheridani* embryo, which was already about 30–40 µm thick, stiff and rigid during the retention in the genital tract. The neonates of *O.* *miranda* and *Z.* *ventriosa* were much larger than the embryos and neonates of *S.* *addisoni* and *R.* *variegata* (Table 1), however, the former taxa has much thinner shells.

### Scanning electron microscopy

**Figure 6:**
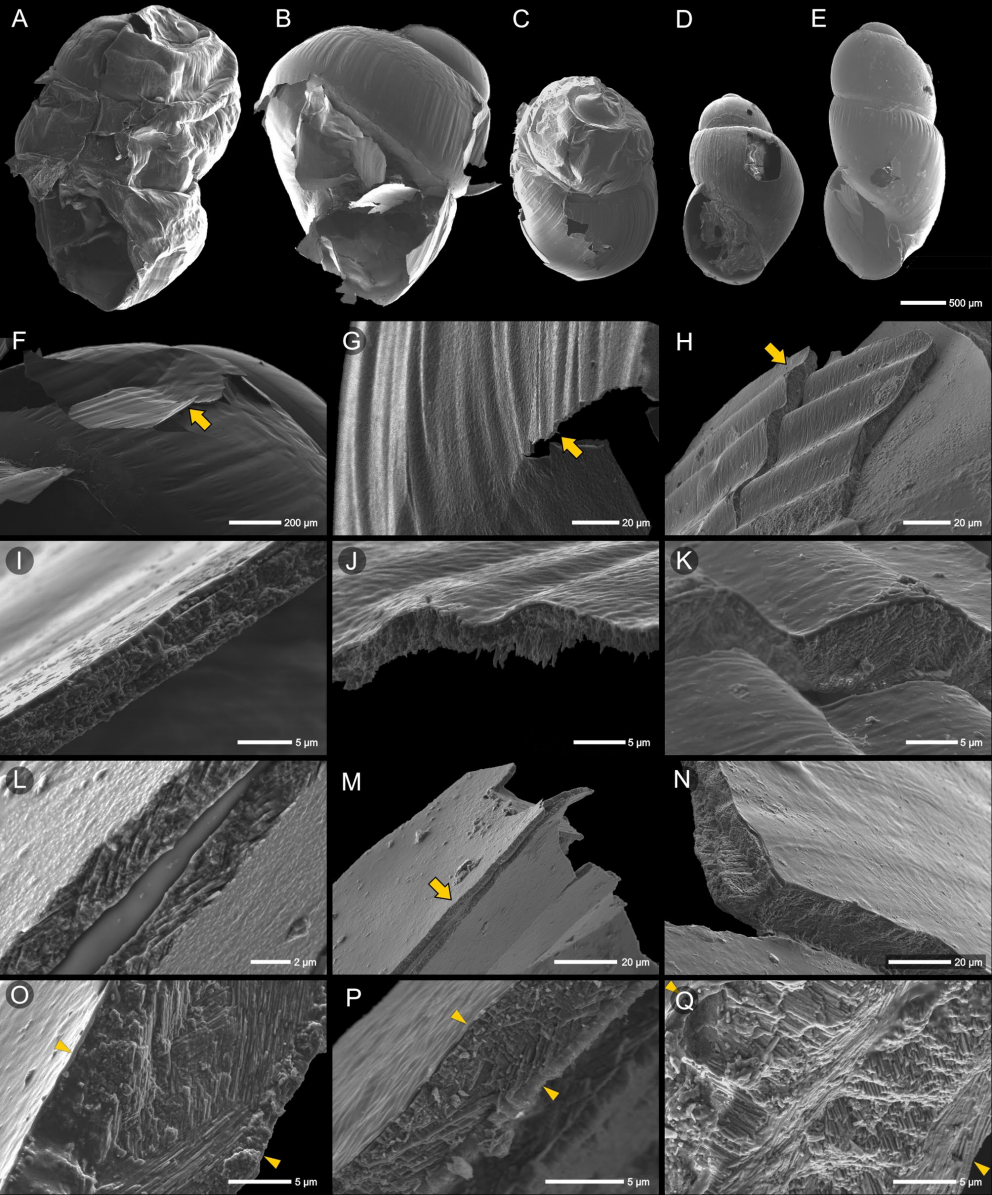
Neonates of *O.* *miranda* (**A,B,F,I,L,M,O**) and *Z.* *ventriosa* (**C,G,J,P**) in direct comparison with hard-shelled embryos and neonates of *R.* *variegata* (**D,N,Q**) and *S.* *addisoni* (**E,H,K**); SEM microphotographs. The vacuum conditions in SEM led to the shrinkage of the thin *O.* *miranda* shell (**A**); freeze-drying of ‘soft-shelled’ neonates prior to SEM imaging reduced the level of deformity (**B,C**). Contrastingly, *R.* *variegata* and *S.* *addisoni* shells do not require special preparation and retain their shape (**D,E**). (**F**) The dented surface of *O.* *miranda* neonate and SEM-close-up (**I**) on a cross-section of the shell just a few micrometers thick (arrow in **F** indicates the region enlarged in **I**). (**G,J**) Shell of *Z.* *ventriosa* in comparison with similarly ornamented fragment of *S.* *addisoni* (**H,K**); note several times thicker shell in the latter (arrows in **G,H** indicate the regions enlarged in **J,K**, respectively). (**L,M**) Inner surface of intact periostracum which still connects two fragments of broken aragonite shell of *O.* *miranda* (the arrow in **M** indicates the region enlarged in **L**); note the difference between shell thickness in *O.* *miranda* (**L,M**) and *R.* *variegata* (**N**). All observed specimens have similar crossed-lamellar microstructure (**L–Q**). However, just as shell thickness, also the number of lamellar layers of alternate orientation within the shell differs (**L,M,O,P** vs **N,Q**).

After the non-invasive microCT scan, we scanned embryos and neonates using SEM (Fig. 6). The different properties of the shells of *Z.* *ventriosa* and *O.* *miranda* vs*.* *S.* *addisoni* and *R.* *variegata* were already visible during the preparation of the analysis. Under vacuum conditions, the soft shells of *Z.* *ventriosa* and *O.* *miranda* shrank and crumpled, creating a cellophane-like surface (Fig. 6A). Embryos and neonates of *S.* *addisoni* and *R.* *variegata* did not require any special preparation and their shell shape remained unchanged under the vacuum conditions applied during the SEM examination (Fig. 6D,E). To reduce the shell deformations, we freeze-dried the next group of thin-shelled neonates prior to SEM analyses (Fig. 6B,C).

The SEM studies allowed for complementary measurements of the shells. In the broken fragments of *Z.* *ventriosa* and *O.* *miranda*, the thickness of the shell wall ranged from 2–3 µm (Fig. 6F,G,I,J,L,M) to 18 µm in the largest neonate of *O.* *miranda* (Fig. 6O). The shells of *S.* *addisoni* (Fig. 6H,K) and *R.* *variegata* (Fig. 6N) are several times thicker.

All analyzed samples have a thin (< 1 µm) layer of periostracum. Beneath that, we recognized the aragonite shells composed of lamellae with alternate orientations (i.e. crossed lamellar microstructure), which is typical for adult gastropods. The crossed lamellar part of the gastropod shell usually includes a few macrolayers of different orientation of lamellae^22^. The number of macrolayers in the ‘soft’ and ‘hard’ embryonic shells varies and possibly influences the thickness of the shell [compare to the thin wall of *O.* *miranda* (Fig. 6L) and the number of alternating macrolayers in *R.* *variegata* (Fig. 6Q)].

### Phylogeny reconstruction

**Figure 7:**
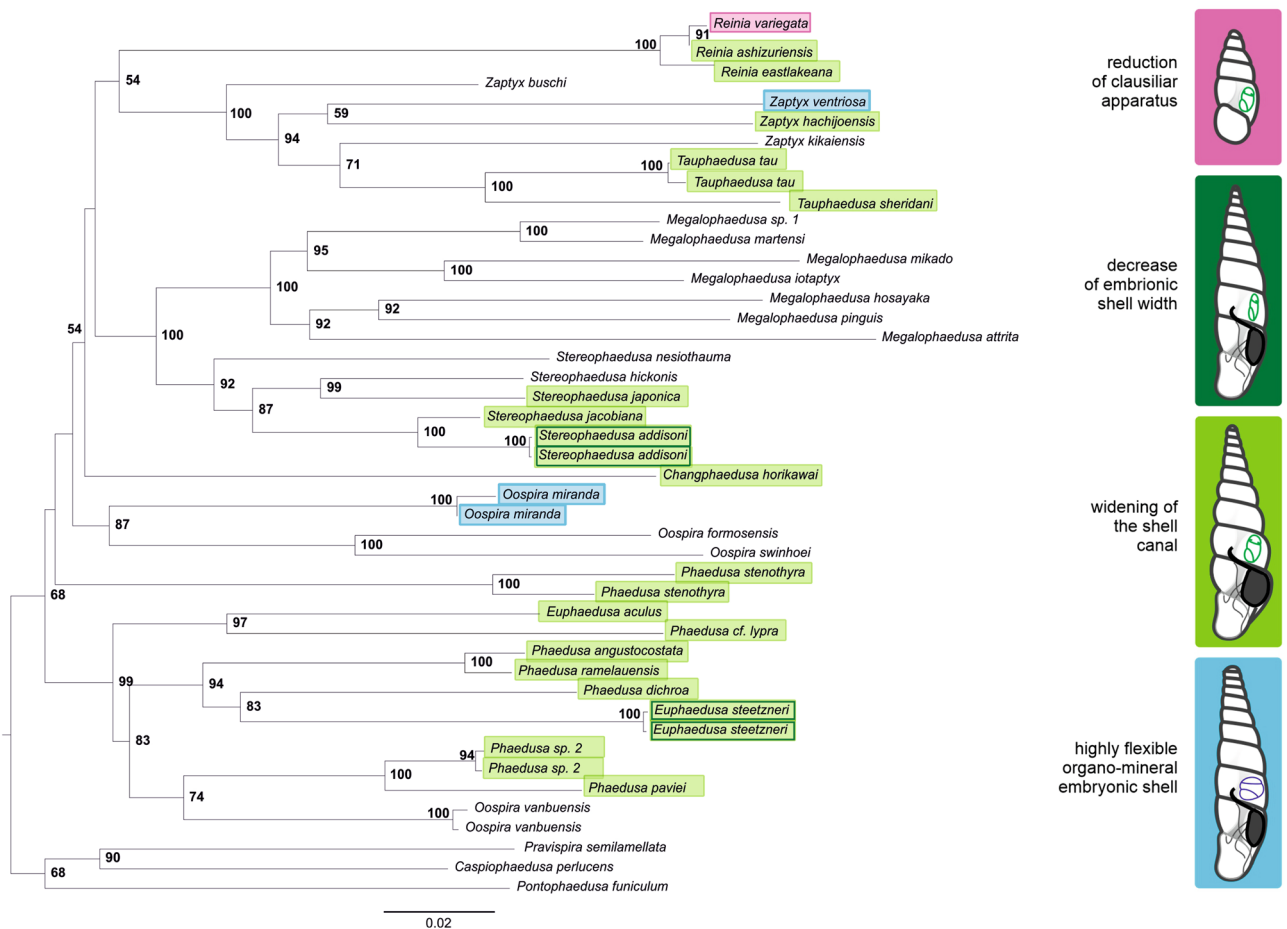
IQ-TREE phylogeny reconstruction of selected Phaedusinae, based on concatenated COI, 16S, 28S, H3 and H4 markers. Viviparous species are indicated with color frame. Four types of adaptations of shell shape and structure recognized in viviparous species presented as schematic drawings of adult shell (based on Nordsieck (2007), modified), with the clausilium plate marked in black.

IQ-TREE phylogeny reconstruction clearly shows that the viviparous strategy occurs independently many times within Phaedusinae (Fig. 7). The development of ‘soft-shelled’ neonates in *O.* *miranda* and *Z.* *ventriosa* cannot be explained by descent of these species from a common ancestor. Similarly, the widening of the shell canal and the decrease in embryonic shell width evolved repeatedly within the studied group. Almost complete reduction of the clausiliar occurred only in *R.* *variegata*.

## Discussion

In this study, we showed that shell morphology and its physical properties resulted from the evolutionary compromise between protection (against potential predators) and giving birth to hard-shelled juveniles. This idea was initially explored for closely related species of the Clausiliinae subfamily, which have a significant increase of shell canal patency in the body whorl of viviparous taxa, in contrast to the narrow passage through apertural barriers in oviparous species^7^. The current study reports the novel finding of unusually flexible embryonic shells, which can pass through the apertural barriers during birth and compares it with adaptations previously recognized in viviparous gastropods.

In the Phaedusinae subfamily, the extreme stage of adaptation to viviparity, with the complete reduction of the clausilium, occurs in *Reinia* *variegata.* According to available phylogenies, *R.* *variegata* belongs to the lineage which includes only viviparous taxa^17,19^, however most of them (e.g., *R.* *ashizurensis*), did not lose the clausilium^23^, and represent less advanced adaptations to viviparity. Reduction of apertural barriers occurred also in members of other clausiliid subfamilies, for example in *Balea* *perversa* L., *Macroptychia* *africana* (Melvill & Ponsonby, 1899) (subfamily Clausiliinae), and *Temesa* *clausilioides* (Reeve, 1849) (Peruiniinae)^14^. This suggests that similar ecological drivers and developmental constraints affect the whole family.

In his taxonomical papers, Nordsieck recognized a modification of apertural barriers including a broad clausilium plate and spirally ascending inferior lamella, as being associated with viviparous reproduction in the Phaedusinae^21^. The occurrence of the broad clausilium plate allows for overcoming the mechanical constraints for passage of the neonate, yet the cited author did not consider it explicitly as an adaptation to viviparity. Broad clausilium plates occur, among others, in species of the genera *Phaedusa*, *Reinia*, *Euphaedusa*, and *Tauphaedusa*. Despite a similar morphology of apertural barriers in these taxa, they are not closely related^17,19^. Evidently, the modifications occurred in response to selective pressure imposed by the reproductive mode, i.e. obstetric selection.

Due to recent studies on diversity of reproductive modes in Phaedusinae, it is known that some taxa with a narrow clausilium plate are also viviparous and produce shelled neonates^20^. This phenomenon, which is contrary to the opinion mentioned above^21^, we explained by the unusual properties of the embryonic shell, i.e., its high flexibility. In general, the gastropod shell is an organo-mineral composite with crossed lamellar microstructure of at least four orders of hierarchy (1st order lamellae are composed of layers of 2nd order lamellae. Each 2nd order lamella is formed of tens of thousands of 3rd order lamellae. The 3rd order lamellae are formed from numerous 4th order particles, surrounded by a thin organic sheath^24^). Nanoindentation tests of the gastropod shells revealed that the combination of aragonite and organic matrix in the crossed lamellar microstructure has several times greater fracture resistance than abiotic aragonite^24–26^. Most likely, the thin organically enriched layer of biomineral composite (Fig. 6I,J,L,M,O,P) that forms the embryonic shells of *O.* *miranda* and *Z.* *ventriosa* may be flexible enough to withstand squeezing through the apertural barriers during birth and not to crack. After parturition, the ‘soft shells’ regain their shape. So, the tight fit between the size of the shell canal and the width of the embryonic shell is mitigated by the unusual properties of the embryonic shell. It appeared that species capable of producing flexible-shelled embryos, successfully overcome the necessity of adapting apertural barriers to parturition and kept the small patency of the shell canal. We suspect that this adaptation remained unnoticed for so long possibly because these unique shell properties disappear shortly after birth. We are going to analyze this process in detail; currently we may estimate that the shell loses its flexibility within a few hours (not few days) after birth. According to the phylogeny reconstructed in our study and other available data^17,19^, *O.* *miranda* and *Z.* *ventriosa* represent two phylogenetically distant lineages within the Phaedusinae, so flexible shelled embryos in these snails must have evolved convergently. Figure 7 visualizes the distribution of species with ’soft shelled’ embryos within the subfamily. In light of this finding, the identification of a viviparous reproductive mode in a particular species based solely on adult shell morphology, for example the width of clausilium, is not feasible.

While our study on obstetric selection gives a new perspective in study of shelled molluscs, researchers have well explored the link between life history evolution, calcification and skeleton morphology in mammals and other vertebrates^1,6,27,28^. Those studies have mainly focused on the sexually dimorphic pelvis^4,29^ and differences in egg-shell properties (brittle-shelled vs. pliable-shelled eggs)^30,31^. Soft-shelled reptile eggs are sensitive to desiccation and physical deformation, but are not prone to getting stuck in a narrow pelvic aperture. In contrast, brittle-shelled eggs cannot undergo deformation to pass through the pelvic aperture. However, the possible constraints resulting from the production of rigid eggs, are mitigated because females might use pelvic kinesis to pass them at oviposition^6,29^. This adaptation closely resembles the increased flexibility of pelvic joints before labour in women and the accompanying malleable fontanelles of newly born human babies^2^. The recent discovery of soft-shelled eggs in Mesozoic dinosaurs and the reconstruction of the ancestral state of the eggshell composition that included all known fossil eggshell types, has revealed that the first dinosaur egg was also soft-shelled, while the calcified eggshell evolved later^32^. Flexible, ’soft-shelled’ embryos in viviparous snails, which we observed in clausiliids, are a similar response to obstetric selection. In ongoing more widespread investigation of shelled embryos in other viviparous gastropod taxa we expect to reveal the wide spectrum of shell calcification.

The repeated evolution of viviparity in clausiliids, which clearly required overcoming the mechanical constraints, implies that this reproductive mode was advantageous under different ecological conditions, even if adaptation to live bearing weakens the protective function of apertural barriers. Retention of developing embryos in the genital tract protects them from many environmental stressors and after birth, juveniles can actively avoid dry or submerged places and seek food immediately. In contrast, egg clutches, if not hidden in protective microhabitats (in soil, under bark), are vulnerable to inundation or desiccation. The proximate factor driving the switch in reproductive mode is still unknown, but it seems that for small and middle-sized, iteroparous gastropods, such as clausiliids, the increase in reproductive success through higher fecundity has not evolved; in contrast, they invested more resources in survival of a single progeny. They achieved this goal by producing larger eggs, better provided with nutrients in oviparous species, or by compromising obstetric selection and protecting embryos in the genital tract.

## Methods

For this study, we have selected eight taxa with viviparous reproduction positively verified by direct observation in the laboratory or by X-rays reported in the previous study^20^. We cultured and bred these species (except *E.* *cylindrella*) for several years at the Department of Invertebrate Zoology and Hydrobiology of the University of Łódź. For their original localities see Table 2; adult shells are presented in Figs. 1A,D, 2A,E,I and 4A,E. The studied snails never laid viable eggs with early stages of embryo development; instead they delivered neonates, which began moving and feeding immediately after birth (live-bearing reproduction).

**Table 2:** Viviparous Phaedusinae species kept in the laboratory: scientific name, number of wild-collected individuals at the start of laboratory colony, original localities and sampling data.

Species name	Number of snails	Original locality: country, region/site, coordinates	Date of sampling	Collector(s)
*Tauphaedusa* *sheridani* (L. Pfeiffer, 1865)	28	Taiwan Keelung City, Dawulun 25.159, 121.709	21-Nov-2015	Shu Ping Wu
*Reinia* *variegata* (A. Adams, 1868)	32	Japan Ogago, Hachijo, Tokyo 33.1019, 139.776	01-Jun-2016	Takumi Saito
*Reinia* *ashizuriensis* Azuma, 1968	14	Japan Kochi, Tosashimizu City, Cape Ashizuri 32.724, 133.013	20-Jun-2016	Yuki Miki Osamu Miura Daishi Yamazaki
*Euphaedusa* *steetzneri* (Pilsbry, 1919)	6	China Sichuan, Wenchuan County 31.4085, 103.532	09-Sep-2018	Takahiro Hirano B. Ye
*Oospira* *miranda* (Loosjes & Loosjes-van Bemmel, 1973)	43	Vietnam Ninh Binh, Cuc Phuong National Park 20.2949, 105.67	05-May-2019	Anna Drozd Thuy Dieu Dinh Katharina v. Oheimb Parm v. Oheimb
*Stereophaedusa* *addisoni* (Pilsbry, 1901)	2	Japan Kagoshima, Kagoshima Shrine, Hayato, Kirishima 31.7534, 130.738	19-Sept-2019	Rei Ueshima Takahiro Asami Anna Drozd
*Zaptyx* *ventriosa* (Schmacker & Boettger, 1891)	15	Taiwan Pingtung County, Manchou Townshio, Gankou Village 21.9926, 120.836	07-Nov-2019	Chung-Chi Hwang

For juvenile shell size and shell thickness measurements, we used newborn snails preserved in ethanol. We photographed the randomly selected neonates/hatchlings using a Leica M205C stereomicroscope, with a Leica DFC 295 camera (Leica Microsystems, Germany), available in the Department of Invertebrate Zoology and Hydrobiology, University of Lodz. Then we measured the height and width of the shell (SH, SW) using the Leica Application Suite V4.5 microscope software.

We examined several individuals of each species anatomically and shelled embryos found in the genital tract were carefully extracted for measurements. For dissection, we used the *niku-nuki* method^33^, allowing for the extraction of soft body parts of the specimen poured beforehand with boiling water, without breaking the shell.

To examine shell structure and thickness, we used microcomputed tomography (microCT) and scanning electron microscopy (SEM). The number of scanned individuals was 2–3 adults and 2–3 juveniles per species.

To preserve embryos and neonates for scanning, we fixed specimens overnight in 2.5% glutaraldehyde solution, then transferred them sequentially to 30%, 50% and 70% ethanol for dehydration^34^. Finally, we embedded them in 0.5 mL eppendorf type tubes (Medlab Products), filled with 0.5% low melting temperature agarose. The agarose gel immobilizes the samples for microCT scans and prevents them from desiccation.

We collected microCT data using the Zeiss XRadia MicroXCT-200 system, equipped with a 90 kV/8 W tungsten X-ray source, in the Laboratory of Microtomography, Institute of Paleobiology, Polish Academy of Sciences, Warsaw, Poland. We reconstructed radial projections using XMReconstructor software, then prepared the virtual sections and 3D visualizations with the XM3DViewer and Avizo Fire software. For the shell thickness measurements of microCT sections, we used XM3DViewer. To compare the shell density of the studied species, we used the same scanning and reconstruction parameters for all specimens. For our purpose, we did not need absolute density values, but only comparability between the scans, so accordingly we use the term ‘relative density’^35^. The supplementary online materials (SOM) provide detailed parameters of the scans.

We conducted scanning electron microscope (SEM) studies at the Institute of Paleobiology, PAS, Warsaw, Poland, using Quattro S (Thermo Fisher Scientific) equipment. We investigated the samples in high vacuum or in low vacuum (50 Pa) mode, in the 2–5 keV accelerating voltage range. Prior to the investigations in high vacuum mode, we coated the samples with a 10 nm carbon layer, using a CCU-010 high vacuum sputter (Safematic). We fixed the samples directly onto the stubs using silver cement. Finally, for freeze-drying (c.a. −20 °C) we used silica gel as a desiccant.

For the evolutionary context of this study, we generated the multigene phylogeny covering Phaedusinae species with known reproductive strategies. We used a subset of the specimens available in BOLD dataset (https://doi.org//10.5883/DS-PHAEDUSA), supplemented with newly obtained sequences of seven new individuals representing four species (*S.* *addisoni*, *E.* *steetzneri*, *O.* *miranda* and *Z.* *ventriosa*), which we isolated following the same procedure^17^. For the purposes of this study COI, 16S, 28S, H3 and H4 markers were amplified and sequenced. The newly obtained sequences and those obtained from previous study^17^, together with all metadata, were uploaded to the publicly available BOLD dataset (DS-SOFTSHEL). See also the supplementary online materials (SOM).

We aligned sequences of each marker using MAFT aligner^36^ with automatic settings. The markers were concatenated and the final dataset was 2322 bp long.

We reconstructed the phylogeny of the concatenated multimarker dataset using the maximum likelihood (ML) approach in IQ-TREE software^37^. We set the substitution model to TVMe + I + G4, following the specification of Bayesian information criterion (BIC) obtained from ModelFinder available through IQ-TREE^38^. We obtained supports using bootstrapping with 1000 replicates.

## Supplementary Information

Supplementary Information.

## Data Availability

GenBank accession codes for the sequences used in this study are provided in the supplementary online materials (SOM). All metadata were uploaded to publicly available BOLD dataset (DS-SOFTSHEL). The microCT data is available upon a reasoned request to the corresponding author.
