# Supplementary material for: Flexible embryonic shell allies large offspring size and anti-predatory protection in viviparous snails
Source: Sci Rep. 2022 Oct 25;12:17881. doi: 10.1038/s41598-022-22651-w (PMC9605993; doi:10.1038/s41598-022-22651-w)
Supplement: Supplementary file 1 — Supplementary Information. [file 41598_2022_22651_MOESM1_ESM.doc]

Supplementary Online Material (SOM)

**MicroCT scans parameters used in this study.**

| **Fig. 1 B,C** |
| --- |
| Images collected with an XRadia MicroXCT-200 imaging system equipped with a 90 kV/8W tungsten X-ray source in the Laboratory of Microtomography, Institute of Paleobiology, Polish Academy of Sciences, Warsaw. |
| The scan was performed using the following parameters: voltage: 60 kV; current: 100 μA; power: 6W; exposure time: 4 s; voxel size: 5.43 μm, 1000 images. |
|  |
| **Fig. 1 E,F** |
| Images collected with an XRadia MicroXCT-200 imaging system equipped with a 90 kV/8W tungsten X-ray source in the Laboratory of Microtomography, Institute of Paleobiology, Polish Academy of Sciences, Warsaw. |
| The scan was performed using the following parameters: voltage: 60 kV; current: 100 μA; power: 6W; exposure time: 4 s; voxel size: 5.43 μm, 1000 images. |
|  |
| **Fig. 3 A,B** |
| Images collected with an XRadia MicroXCT-200 imaging system equipped with a 90 kV/8W tungsten X-ray source in the Laboratory of Microtomography, Institute of Paleobiology, Polish Academy of Sciences, Warsaw. |
| The scan was performed using the following parameters: voltage: 60 kV; current: 100 μA; power: 6W; exposure time: 4 s; voxel size: 5.18 μm, 1000 images. |
|  |
| **Fig. 3 C** |
| Images collected with an XRadia MicroXCT-200 imaging system equipped with a 90 kV/8W tungsten X-ray source in the Laboratory of Microtomography, Institute of Paleobiology, Polish Academy of Sciences, Warsaw. |
| The scan was performed using the following parameters: voltage: 60 kV; current: 100 μA; power: 6W; exposure time: 5 s; voxel size: 5.43 μm, 1000 images. |
|  |
| **Fig. 3 D** |
| Images collected with an XRadia MicroXCT-200 imaging system equipped with a 90 kV/8W tungsten X-ray source in the Laboratory of Microtomography, Institute of Paleobiology, Polish Academy of Sciences, Warsaw. |
| The scan was performed using the following parameters: voltage: 60 kV; current: 100 μA; power: 6W; exposure time: 4 s; voxel size: 5.43 μm, 1000 images. |
|  |
| **Fig. 3 E,F** |
| Images collected with an GE Phoenix v|tome|x s, X-ray Microtomography Lab, Faculty of Science and Technology, University of Silesia, Katowice, Poland. |
| The scan was performed using the following parameters: voltage: 150 kV; current: 70 μA; exposure time: 250 ms; voxel size: 10 μm, 1200 images. |
| **Fig. 3 G** |
| Images collected with an GE Phoenix v|tome|x s, X-ray Microtomography Lab, Faculty of Science and Technology, University of Silesia, Katowice, Poland. |
| The scan was performed using the following parameters: voltage: 120 kV; current: 80 μA; exposure time: 500 ms; voxel size: 15 μm, 1000 images. |
|  |
| **Fig. 3 H** |
| Images collected with an XRadia MicroXCT-200 imaging system equipped with a 90 kV/8W tungsten X-ray source in the Laboratory of Microtomography, Institute of Paleobiology, Polish Academy of Sciences, Warsaw. |
| The scan was performed using the following parameters: voltage: 45 kV; current: 133 μA; power: 6W; exposure time: 12 s; voxel size: 17.04 μm, 1600 images. |
|  |
| **Fig. 5 A,G** |
| Images collected with an XRadia MicroXCT-200 imaging system equipped with a 90 kV/8W tungsten X-ray source in the Laboratory of Microtomography, Institute of Paleobiology, Polish Academy of Sciences, Warsaw. |
| The scan was performed using the following parameters: voltage: 20 kV; current: 200 μA; power: 4W; exposure time: 40 s; voxel size: 3.93 μm, 1600 images. |
|  |
| **Fig. 5 B,H** |
| Images collected with an XRadia MicroXCT-200 imaging system equipped with a 90 kV/8W tungsten X-ray source in the Laboratory of Microtomography, Institute of Paleobiology, Polish Academy of Sciences, Warsaw. |
| The scan was performed using the following parameters: voltage: 30 kV; current: 133 μA; power: 4W; exposure time: 6 s; voxel size: 4.33 μm, 1600 images. |
|  |
| **Fig. 5 C,I** |
| Images collected with an XRadia MicroXCT-200 imaging system equipped with a 90 kV/8W tungsten X-ray source in the Laboratory of Microtomography, Institute of Paleobiology, Polish Academy of Sciences, Warsaw. |
| The scan was performed using the following parameters: voltage: 35 kV; current: 140 μA; power: 5W; exposure time: 6 s; voxel size: 4.45 μm, 1600 images. |
|  |
| **Fig. 5 D,J** |
| Images collected with an XRadia MicroXCT-200 imaging system equipped with a 90 kV/8W tungsten X-ray source in the Laboratory of Microtomography, Institute of Paleobiology, Polish Academy of Sciences, Warsaw. |
| The scan was performed using the following parameters: voltage: 30 kV; current: 133 μA; power: 4W; exposure time: 15 s; voxel size: 5.29 μm, 1600 images. |
|  |
| **Fig. 5 E,K** |
| Images collected with an XRadia MicroXCT-200 imaging system equipped with a 90 kV/8W tungsten X-ray source in the Laboratory of Microtomography, Institute of Paleobiology, Polish Academy of Sciences, Warsaw. |
| The scan was performed using the following parameters: voltage: 30 kV; current: 133 μA; power: 4W; exposure time: 15 s; voxel size: 5.29 μm, 1600 images. |
|  |
| **Fig. 5 F,L** |
| Images collected with an XRadia MicroXCT-200 imaging system equipped with a 90 kV/8W tungsten X-ray source in the Laboratory of Microtomography, Institute of Paleobiology, Polish Academy of Sciences, Warsaw. |
| The scan was performed using the following parameters: voltage: 30 kV; current: 133 μA; power: 4W; exposure time: 7 s; voxel size: 3.37 μm, 1600 images. |
|  |
| **Fig. 5 P** |
| Images collected with an XRadia MicroXCT-200 imaging system equipped with a 90 kV/8W tungsten X-ray source in the Laboratory of Microtomography, Institute of Paleobiology, Polish Academy of Sciences, Warsaw. |
| The scan was performed using the following parameters: voltage: 35 kV; current: 140 μA; power: 5W; exposure time: 6 s; voxel size: 4.45 μm, 1600 images. |

**GenBank accession numbers for nucleotide sequences analysed in this study.**

|  | Newly obtained | From Mamos et al. (2021) |
| --- | --- | --- |
| COI | OP494296 - OP494302 | MW378728, MW378729, MW378732, MW378734, MW378738, MW378740, MW378745, MW378749, MW378750, MW378753, MW378755, MW378756, MW378760 - MW378763, MW378767, MW378769, MW378772, MW378774, MW378777, MW378779, MW378784, MW378788, MW378797, MW378800, MW378810, MW378812, MW378816, MW378818, MW378831, MW378837, MW378842, MW378845, MW378854, MW378856, MW378857 |
| 28S | OP442948 - OP442954 | MW379092, MW379093, MW379095, MW379097, MW379101, MW379103, MW379106, MW379110, MW379111, MW379113, MW379115, MW379116, MW379119, MW379121 ­- MW379123, MW379126, MW379128, MW379130, MW379131, MW379133, MW379135, MW379140, MW379143, MW379147, MW379149, MW379155, MW379157, MW379160, MW379162, MW379167, MW379170, MW379172, MW379174, MW379177, MW379183, MW379185, MW379186 |
| 16S | OP494304 - OP494308 | MW378862, MW378863, MW378866, MW378868, MW378872, MW378874, MW378879, MW378883, MW378884, MW378886, MW378888, MW378889, MW378894, MW378896 - MW378898, MW378902, MW378904, MW378906, MW378907, MW378910, MW378912, MW378917, MW378921, MW378930, MW378933, MW378941, MW378943, MW378947, MW378949, MW378959, MW378965, MW378968, MW378971, MW378974, MW378980, MW378982, MW378983 |
| H3 | OP538564 - OP538568 | MW378990, MW378991, MW378993, MW378995, MW379001, MW379004, MW379008, MW379009, MW379011, MW379013, MW379014, MW379017, MW379019 - MW379021, MW379024, MW379026, MW379028, MW379029, MW379032, MW379033, MW379037, MW379041, MW379046, MW379049, MW379055, MW379057, MW379060, MW379062, MW379067, MW379071, MW379074, MW379077, MW379080, MW379086, MW379087 |
| H4 | OP494337 - OP494339 | MW379188, MW379189, MW379192, MW379193, MW379197, MW379199, MW379201, MW379203, MW379204, MW379207 - MW379209, MW379211, MW379212, MW379221, MW379225, MW379226, MW379231, MW379233, MW379238, MW379241, MW379244, MW379246, MW379248, MW379252, MW379253 |
